# Supplementary material for: A high-throughput neutralizing antibody assay for COVID-19 diagnosis and vaccine evaluation
Source: Nat Commun. 2020 Aug 13;11:4059. doi: 10.1038/s41467-020-17892-0 (PMC7426916; doi:10.1038/s41467-020-17892-0)
Supplement: Supplementary file 1 — Supplementary Information [file 41467_2020_17892_MOESM1_ESM.pdf]

Supplementary Methods

Standard operation procedure (SOP) for mNeonGreen SARS-CoV-2 Neutralization assay

<u>Reagents and equipment</u>
- Greiner Bio-One™ CellStar™ 96-Well, Cell Culture-Treated, Flat-Bottom (Greiner Bio-One™ 655090)
-Corning™ Clear Polystyrene 96-Well Microplates, round bottom (Corning Cat. No: 3799)
-Culture medium: DMEM (Gibco Cat. No: 11965) supplemented with 10% FBS, 1%p/S
-Assay medium: phenol red-free DMEM (Gibco Cat.No: 31053028), supplemented with 2% GlutaMAX (Gibco, Cat. No: 35050079), 2% FBS, 1%P/S.
-Fetal Bovine Serum (Hyclone Cat. No.: SH30071)
-Penicillium-Streptomycin (P/S) (10,000 U/ml) (Gibco Cat. No: 15140122)
-Gibco™ Trysin-EDTA (0.25%), phenol red (Gibco Cat. No.: 25200072)
-Phosphate Buffered Saline (PBS) solution, pH 7.4 (Gibco Cat. No.: 10010049)
-Hoechst 33342 Solution (ThermoFisher Scientific, Cat No: 62249)
-Reagent Reservoirs 25 ml (Gilson Cat. No.: F267660) & 50 ml (Gilson Cat. No.: F267670)
-Cell Counting slides for TC10™/TC20™ Cell Counter, dual-chamber (Bio-Rad Cat. No.: 1450011)
-Corning™ Cell Culture Treated flasks (Corning™ Cat. No.: 431080)
-VACUBOY Hand Operator (INTEGRA Biosciences)
-Eppendorf Xplorer pipette, electronic 12 channel pipette 15-300 µL (Eppendorf Cat. No.: 4861000031)
-TC20™ Automated Cell Counter (Bio-Rad)
-Tissue culture CO_2 incubator
- Cytation™ 7 Cell Imaging Multi-Mode Reader (BioTek)

<u>Prepare cells prior to infection (on day 0)</u>

Seed cells into 96-well plate Solid Black Polystyrene Microplates with clear bottom.

- Cells are grown a T-175 flask. Upon seeing cells, remove the medium from the cells using the VACUBOY.
- Briefly rinse the cell layer with 25 ml PBS to remove all traces of serum. Remove PBS using the VACUBOY. Repeat the PBS wash once.
  Note: make sure rinse every corner of the flask to get rid of any trypsin inhibitor.
- Add 3 ml Trypsin-EDTA solution. Observe cells under an inverted microscope until cell layer is dispersed (usually within 2 minutes at room temperature).
- Tap the flask vigorously to detach cells, add 12 ml of complete growth medium and pipet up and down gently to disperse the cell suspension. Transfer the cell suspension into a 50-ml falcon tube.
- Centrifuge at room temperature for 3 min at 1,200×rpm (300g).
- Remove media completely.
- Resuspend cells in 10 ml assay medium. Disperse cells by pipetting up and down.
- Count the cell number using the cell counter (C-Chip DHC-N01-5). Count live cells by mixing 50 µl of trypan blue with 50 µl of cell suspension.
- Dilute cells to a final concentration of 2.4×10^5 cells/ml. Add 50 µl of the diluted cell suspension to each well of a 96-well plate to reach 1.2×10^4 cells/well.
- Incubate the plates at 37°C with 5% CO_2.

<u>Prepare serum dilutions and infection (on day 1)</u>

- Heat inactivate all the sera at 56°C for 30 min.
- Prepare serial dilutions of serum in a round bottom 96-well plate. Prepare 10 serials of 2-fold dilutions. The highest dilution is 10-folds. Prepare dilutions in duplicates. See the diagram below.

D1	D2	D3	D4	D5	D6	D7	D8	D9	D10
10	20	40	80	160	320	640	1280	2156	0

D1: 6 µl serum + 54 µl of assay medium; D2-D9: 30 µl diluted samples + 30 µl assay medium. D10: 30 µl assay medium

- Transfer 30 µl from column #D1 to column #D2 using the electronic pipets with settings of P/M, 30/50 and speed 6/4, 3X. Repeat the step for columns D3 to D9.
- At dilution D9, take 30 µl from after dilution and discard. The total volume of the dilute serum should be 30 µl/well.
- Sera dilution plate set-up. Samples are run in duplicates.

		1	2	3	4	5	6	7	8	9	10	11	12
	A												
Sample 1	B		(+)UN	1:20	1:40	1:80	1:160	1:320	1:640	1:1280	1:2560	(-)cells	
Sample 1	C		(+)UN	1:20	1:40	1:80	1:160	1:320	1:640	1:1280	1:2560	(-)cells	
Sample 2	D		(+)UN	1:20	1:40	1:80	1:160	1:320	1:640	1:1280	1:2560	(-)cells	
Sample 2	E		(+)UN	1:20	1:40	1:80	1:160	1:320	1:640	1:1280	1:2560	(-)cells	
Sample 3	F		(+)UN	1:20	1:40	1:80	1:160	1:320	1:640	1:1280	1:2560	(-)cells	
Sample 3	G		(+)UN	1:20	1:40	1:80	1:160	1:320	1:640	1:1280	1:2560	(-)cells	
	H												

- In BSL-3, add 30 µl of diluted SARS-CoV2-mNG virus (MOI is 0.5) to each well of the serum. Mix the serum with virus solutions thoroughly by gentle pipetting.
- Incubate the plates at 37°C for 1 hour.
- Transfer 50 µl virus-serum complexes to the 96-well plates seeded on day -1 (cell plates containing 50 µl of culture media per well, after adding the diluted reporter virus the total volume of each well will be 100 µl). Mix using an electronic 12-channel pipette (setting, P/M, 50/75, speed 3/3, 2X).
  Example: cells+Ab+reporter virus plate setup (black plate)

		1	2	3	4	5	6	7	8	9	10	11	12
	A												
Sample 1	B		+V	+V	+V	+V	+V	+V	+V	+V	+V	+V	No virus
Sample 1	C		+V	+V	+V	+V	+V	+V	+V	+V	+V	+V	No virus
Sample 2	D		+V	+V	+V	+V	+V	+V	+V	+V	+V	+V	No virus
Sample 2	E		+V	+V	+V	+V	+V	+V	+V	+V	+V	+V	No virus
Sample 3	F		+V	+V	+V	+V	+V	+V	+V	+V	+V	+V	No virus
Sample 3	G		+V	+V	+V	+V	+V	+V	+V	+V	+V	+V	No virus
	H												

- Incubate at 37°C for 20 hr.

Data acquisition and analysis (on day 2).

- At 20 h post-infection, in BSL-3 facility, add 25 µl diluted Hoechst 33342 Solution (diluted at 400x in PBS) to each well of the 96-well plate.
- After incubating at 15 min at 37°C, acquire the images with both DAPI staining (in blue) and mNG signals (green) using Cytation™ 7 Cell Imaging Multi-Mode Reader (BioTek) according to the manufacturer's instructions.
  1) Count the total cell numbers: mean intensities (in blue) within the primary mask ≥5000.
  2) Count the mNG-positive cells: mean intensities (in green) within the secondary mask ≥3100.
     The threshold of green intensity was selected to distinguish mNG-positive signals from the background.
  3) Determine the infection rate: (100×mNG-positive cell number/total cell number)%.
     Optimization may be required to achieve the infection rate in the no-serum controls at 10%-30% for robustness. The cell controls (without viruses) should be <1%.

- Plot the neutralization values in Prism to calculate the NT_{50}s and Hislopes.
  1) Normalize the infection rate to the no-serum control wells.

2) Plot the relative infection rate versus the dilution (log10 values) in the Prism software 8.3.
3) Fit the curve and calculate the Neutralizing titers (NT_{50}) using the nonlinear regression model: log(inhibitor) vs. response-variable slope (four parameters) with constrain of bottom to 0 and top to 100.
4) Data interpretation.
   $NT_{50}<20$: negative; $NT_{50}\geq 20$: Positive

Supplementary Figure 1

attaaaggtttataccttcccaggtaacaaaccaaccaactttcgatctcttgtagatctgttctctaaacgaactttaaaatctgtgtggctgtcactcggctgcatgcttagt
gcactcacgcagtataattaataactaattactgtcgttgacaggacacgagtaactcgtctatcttctgcaggctgcttacggtttcgtccgtgttgcagccgatcatcag
cacatctaggtttcgtccgggtgtgaccgaaaggtaagatggagagccttgtccctggtttcaacgagaaaacacacgtccaactcagtttgcctgttttacaggttcgc
gacgtgctcgtacgtggctttggagactccgtggaggaggtcttatcagaggcacgtcaacatcttaaagatggcacttgtggcttagtagaagttgaaaaaggcgtttt
gcctcaacttgaacagccctatgtgttcatcaaacgttcggatgctcgaactgcacctcatggtcatgttatggttgagctggtagcagaactcgaaggcattcagtacg
gtcgtagtggtgagacacttggtgtccttgtccctcatgtgggcgaaataccagtggcttaccgcaaggttcttcttcgtaagaacggtaataaaggagctggtggccat
agttacggcgccgatctaaagtcatttgacttaggcgacgagcttggcactgatccttatgaagattttcaagaaaactggaacactaaacatagcagtggtgttacccg
tgaactcatgcgtgagcttaacggaggggcatacactcgctatgtcgataacaacttctgtggccctgatggctaccctcttgagtgcattaaagaccttctagcacgtg
ctggtaaagcttcatgcactttgtccgaacaactggactttattgacactaagaggggtgtatactgctgccgtgaacatgagcatgaaattgcttggtacacggaacgtt
ctgaaaagagctatgaattgcagacaccttttgaaattaaattggcaaagaaatttgacaccttcaatggggaatgtccaaattttgtatttcccttaaattccataatcaaga
ctattcaaccaagggttgaaaagaaaaagcttgatggctttatgggtagaattcgatctgtctatccagttgcgtcaccaaatgaatgcaaccaaatgtgcctttcaactct
catgaagtgtgatcattgtggtgaaacttcatggcagacgggcgattttgttaaagccacttgcgaattttgtggcactgagaatttgactaaagaaggtgccactacttgt
ggttacttaccccaaaatgctgttgttaaaatttattgtccagcatgtcacaattcagaagtaggacctgagcatagtcttgccgaataccataatgaatctggcttgaaaa
ccattcttcgtaagggtggtcgcactattgcctttggaggctgtgtgttctcttatgttggttgccataacaagtgtgcctattgggttccacgtgctagcgctaacataggtt
gtaaccatacaggtgttgttggagaaggttccgaaggtcttaatgacaaccttcttgaaatactccaaaaagagaaagtcaacatcaatattgttggtgactttaaacttaa
tgaagagatcgccattattttggcatctttttctgcttccacaagtgcttttgtggaaactgtgaaaggtttggattataaagcattcaaacaaattgttgaatcctgtggtaatt
ttaaagttacaaaaggaaaagctaaaaaaggtgcctggaatattggtgaacagaaatcaatactgagtcctctttatgcatttgcatcagaggctgctcgtgttgtacgat
caattttctcccgcactcttgaaactgctcaaaattctgtgcgtgttttacagaaggccgctataacaatactagatggaatttcacagtattcactgagactcattgatgcta
tgatgttcacatctgatttggctactaacaatctagttgtaatggcctacattacaggtggtgttgttcagttgacttcgcagtggctaactaacatctttggcactgtttatga
aaaactcaaacccgtccttgattggcttgaagagaagtttaaggaaggtgtagagtttcttagagacggttgggaaattgttaaatttatctcaacctgtgcttgtgaaattg
tcggtggacaaattgtcacctgtgcaaaggaaattaaggagagtgttcagacattctttaagcttgtaaataaatttttggctttgtgtgctgactctatcattattggtggag
ctaaacttaaagccttgaatttaggtgaaacatttgtcacgcactcaaagggattgtacagaaagtgtgttaaatccagagaagaaactggcctactcatgcctctaaaa
gccccaaaagaaattatcttcttagagggagaaacacttcccacagaagtgttaacagaggaagttgtcttgaaaactggtgatttacaaccattagaacaacctactag
tgaagctgttgaagctccattggttggtacaccagtttgtattaacgggcttatgttgctcgaaatcaaagacacagaaaagtactgtgcccttgcacctaatatgatggta
acaaacaataccttcacactcaaaggcggtgcaccaacaaaggttacttttggtgatgacactgtgatagaagtgcaaggttacaagagtgtgaatatcacttttgaactt
gatgaaaggattgataaagtacttaatgagaagtgctctgcctatacagttgaactcggtacagaagtaaatgagttcgcctgtgttgtggcagatgctgtcataaaaact
ttgcaaccagtatctgaattacttacaccactgggcattgatttagatgagtggagtatggctacatactacttatttgatgagtctggtgagtttaaattggcttcacatatgt
attgttctttctaccctccagatgaggatgaagaagaaggtgattgtgaagaagaagagtttgagccatcaactcaatatgagtatggtactgaagatgattaccaaggt
aaacctttggaatttggtgccacttctgctgctcttcaacctgaagaagagcaagaagaagattggttagatgatgatagtcaacaaactgttggtcaacaagacggca
gtgaggacaatcagacaactactattcaaacaattgttgaggttcaacctcaattagagatggaacttacaccagttgttcagactattgaagtgaatagttttagtggttat
ttaaaacttactgacaatgtatacattaaaaatgcagacattgtggaagaagctaaaaaggtaaaaccaacagtggttgttaatgcagccaatgtttaccttaaacatgga
ggaggtgttgcaggagccttaaataaggctactaacaatgccatgcaagttgaatctgatgattacatagctactaatggaccacttaaagtgggtggtagttgtgtttta
agcggacacaatcttgctaaacactgtcttcatgttgtcggcccaaatgttaacaaaggtgaagacattcaacttcttaagagtgcttatgaaaattttaatcagcacgaag
ttctacttgcaccattattatcagctggtatttttggtgctgaccctatacattctttaagagtttgtgtagatactgttcgcacaaatgtctacttagctgtctttgataaaaatct
ctatgacaaacttgtttcaagctttttggaaatgaagagtgaaaagcaagttgaacaaaagatcgctgagattcctaaagaggaagttaagccatttataactgaaagtaa
accttcagttgaacagagaaaacaagatgataagaaaatcaaagcttgtgttgaagaagttacaacaactctggaagaaactaagttcctcacagaaaacttgttacttt
atattgacattaatggcaatcttcatccagattctgccactcttgttagtgacattgacatcactttcttaaagaaagatgctccatatatagtgggtgatgttgttcaagagg
gtgttttaactgctgtggttatacctactaaaaaggctggtggcactactgaaatgctagcgaaagctttgagaaaagtgccaacagacaattatataaccacttacccgg
gtcagggtttaaatggttacactgtagaggaggcaaagacagtgcttaaaaagtgtaaaagtgccttttacattctaccatctattatctctaatgagaagcaagaaattctt
ggaactgtttcttggaatttgcgagaaatgcttgcacatgcagaagaaacacgcaaattaatgcctgtctgtgtggaaactaaagccatagtttcaactatacagcgtaaa
tataagggtattaaaatacaagagggtgtggttgattatggtgctagattttacttttacaccagtaaaacaactgtagcgtcacttatcaacacacttaacgatctaaatga
aactcttgttacaatgccacttggctatgtaacacatggcttaaatttggaagaagctgctcggtatatgagatctctcaaagtgccagctacagtttctgtttcttcacctga
tgctgttacagcgtataatggttatcttacttcttcttctaaaacacctgaagaacattttattgaaaccatctcacttgctggttcctataaagattggtcctattctggacaatc
tacacaactaggtatagaatttcttaagagaggtgataaaagtgtatattacactagtaatcctaccacattccacctagatggtgaagttatcacctttgacaatcttaaga
cacttctttctttgagagaagtgaggactattaaggtgtttacaacagtagacaacattaacctccacacgcaagttgtggacatgtcaatgacatatggacaacagtttg
gtccaacttatttggatggagctgatgttactaaaataaaacctcataattcacatgaaggtaaaacattttatgttttacctaatgatgacactctacgtgttgaggcttttga
gtactaccacacaactgatcctagttttctgggtaggtacatgtcagcattaaatcacactaaaaagtggaaatacccacaagttaatggtttaacttctattaaatgggca
gataacaactgttatcttgccactgcattgttaacactccaacaaatagagttgaagtttaatccacctgctctacaagatgcttattacagagcaagggctggtgaagctg
ctaacttttgtgcacttatcttagcctactgtaataagacagtaggtgagttaggtgatgttagagaaacaatgagttacttgtttcaacatgccaatttagattcttgcaaaa

gagtcttgaacgtggtgtgtaaaacttgtggacaacagcagacaacccttaagggtgtagaagctgttatgtacatgggcacactttcttatgaacaatttaagaaaggt
gttcagataccttgtacgtgtggtaaacaagctacaaaatatctagtacaacaggagtcaccttttgttatgatgtcagcaccacctgctcagtatgaacttaagcatggta
catttacttgtgctagtgagtacactggtaattaccagtgtggtcactataaacatataacttctaaagaaactttgtattgcatagacggtgctttacttacaaagtcctcag
aatacaaaggtcctattacggatgttttctacaaagaaaacagttacacaacaaccataaaaccagttacttataaattggatggtgttgtttgtacagaaattgaccctaa
gttggacaattattataagaaagacaattcttatttcacagagcaaccaattgatcttgtaccaaaccaaccatatccaaacgcaagcttcgataattttaagtttgtatgtga
taatatcaaatttgctgatgatttaaaccagttaactggttataagaaacctgcttcaagagagcttaaagttacatttttccctgacttaaatggtgatgtggtggctattgatt
ataaacactacacaccctcttttaagaaaggagctaaattgttacataaacctattgtttggcatgttaacaatgcaactaataaagccacgtataaaccaaatacctggtg
tatacgttgtctttggagcacaaaaccagttgaaacatcaaattcgtttgatgtactgaagtcagaggacgcgcagggaatggataatcttgcctgcgaagatctaaaac
cagtctctgaagaagtagtggaaaatcctaccatacagaaagacgttcttgagtgtaatgtgaaaactaccgaagttgtaggagacattatacttaaaccagcaaataat
agtttaaaaattacagaagaggttggccacacagatctaatggctgcttatgtagacaattctagtcttactattaagaaacctaatgaattatctagagtattaggtttgaaa
acccttgctactcatggtttagctgctgttaatagtgtcccttgggatactatagctaattatgctaagccttttcttaacaaagttgttagtacaactactaacatagttacacg
gtgtttaaaccgtgtttgtactaattatatgccttatttctttactttattgctacaattgtgtacttttactagaagtacaaattctagaattaaagcatctatgccgactactatag
caaagaatactgttaagagtgtcggtaaattttgtctagaggcttcatttaattatttgaagtcacctaatttttctaaactgataaatattataatttggtttttactattaagtgttt
gcctaggttctttaatctactcaaccgctgctttaggtgttttaatgtctaatttaggcatgccttcttactgtactggttacagagaaggctatttgaactctactaatgtcacta
ttgcaacctactgtactggttctataccttgtagtgtttgtcttagtggtttagattctttagacacctatccttctttagaaactatacaaattaccatttcatcttttaaatgggatt
taactgcttttggcttagttgcagagtggtttttggcatatattcttttcactaggtttttctatgtacttggattggctgcaatcatgcaattgtttttcagctattttgcagtacattt
tattagtaattcttggcttatgtggttaataattaatcttgtacaaatggccccgatttcagctatggttagaatgtacatcttctttgcatcattttattatgtatggaaaagttatg
tgcatgttgtagacggttgtaattcatcTacAtgtatgatgtgttacaaacgtaatagagcaacaagagtcgaatgtacaactattgttaatggtgttagaaggtccttttat
gtctatgctaatggaggtaaaggcttttgcaaactacacaattggaattgtgttaattgtgatacattctgtgctggtagtacatttattagtgatgaagttgcgagagacttg
tcactacagtttaaaagaccaataaatcctactgaccagtcttcttacatcgttgatagtgttacagtgaagaatggttccatccatctttactttgataaagctggtcaaaag
acttatgaaagacattctctctctcatttgttaacttagacaacctgagagctaataacactaaaggttcattgcctattaatgttatagtttttgatggtaaatcaaaatgtga
agaatcatctgcaaaatcagcgtctgtttactacagtcagcttatgtgtcaacctatactgttactagatcaggcattagtgtctgatgttggtgatagtgcggaagttgcag
ttaaaatgtttgatgcttacgttaatacgttttcatcaacttttaacgtaccaatggaaaaactcaaaacactagttgcaactgcagaagctgaacttgcaaagaatgtgtcct
tagacaatgtcttatctacttttatttcagcagctcggcaagggtttgttgattcagatgtagaaactaaagatgttgttgaatgtcttaaattgtcacatcaatctgacataga
agttactggcgatagttgtaataactatatgctcacctataacaaagttgaaaacatgacaccccgtgaccttggtgcttgtattgactgtagtgcgcgtcatattaatgcg
caggtagcaaaaagtcacaacattgctttgatatggaacgttaaagatttcatgtcattgtctgaacaactacgaaaacaaatacgtagtgctgctaaaaagaataactta
ccttttaagttgacatgtgcaactactagacaagttgttaatgttgtaacaacaaagatagcacttaagggtggtaaaattgttaataattggttgaagcagttaattaaagtt
acacttgtgttcctttttgttgctgctattttctatttaataacacctgttcatgtcatgtctaaacatactgacttttcaagtgaaatcataggatacaaggctattgatggtggtg
tcactcgtgacatagcatctacagatacttgttttgctaacaaacatgctgattttgacacatggtttagTcagcgtggtggtagttatactaatgacaaagcttgcccattg
attgctgcagtcataacaagagaagtgggttttgtcgtgcctggtttgcctggcacgatattacgcacaactaatggtgactttttgcatttcttacctagagtttttagtgca
gttggtaacatctgttacacaccatcaaaacttatagagtacactgactttgcaacatcagcttgtgttttggctgctgaatgtacaatttttaaagatgcttctggtaagcca
gtaccatattgttatgataccaatgtactagaaggttctgttgcttatgaaagtttacgccctgacacacgttatgtgctcatggatggctctattattcaatttcctaacaccta
ccttgaaggttctgttagagtggtaacaacttttgattctgagtactgtaggcacggcacttgtgaaagatcagaagctggtgtttgtgtatctactagtggtagatgggta
cttaacaatgattattacagatctttaccaggagttttctgtggtgtagatgctgtaaatttacttactaatatgtttacaccactaattcaacctattggtgctttggacatatca
gcatctatagtagctggtggtattgtagctatcgtagtaacatgccttgcctactattttatgaggtttagaagagcttttggtgaatacagtcatgtagttgcctttaatacttt
actattccttatgtcattcactgtactctgtttaacaccagtttactcattcttacctggtgtttattctgttatttacttgtacttgacattttatcttactaatgatgtttctttttagca
catattcagtggatggttatgttcacacctttagtacctttctggataacaattgcttatatcatttgtatttccacaaagcatttctattggttctttagtaattacctaaagagac
gtgtagtctttaatggtgtttcctttagtacttttgaagaagctgcgctgtgcacctttttgttaaataaagaaatgtatctaaagttgcgtagtgatgtgctattacctcttacgc
aatataatagatacttagctctttataataagtacaagtattttagtggagcaatggatacaactagctacagagaagctgcttgttgtcatctcgcaaaggctctcaatgac
ttcagtaactcaggttctgatgttctttaccaaccaccacaaacctctatcacctcagctgttttgcagagtggttttagaaaaatggcattcccatctggtaaagttgaggg
ttgtatggtacaagtaacttgtggtacaactacacttaacggtctttggcttgatgacgtagtttactgtccaagacatgtgatctgcacctctgaagacatgcttaacccta
attatgaagatttactcattcgtaagtctaatcataatttcttggtacaggctggtaatgttcaactcagggttattggacattctatgcaaaattgtgtacttaagcttaaggtt
gatacagccaatcctaagacacctaagtataagtttgttcgcattcaaccaggacagactttttcagtgttagcttgttacaatggttcaccatctggtgtttaccaatgtgct
atgaggcccaatttcactattaagggttcattccttaatggttcatgtggtagtgttggttttaacatagattatgactgtgtctctttttgttacatgcaccatatggaattacca
actggagttcatgctggcacagacttagaaggtaacttttatggaccttttgttgacaggcaaacagcacaagcagctggtacggacacaactattacagttaatgtttta
gcttggttgtacgctgctgttataaatggagacaggtggtttctcaatcgatttaccacaactcttaatgactttaaccttgtggctatgaagtacaattatgaacctctaaca
caagaccatgttgacatactaggacctctttctgctcaaactggaattgccgttttagatatgtgtgcttcattaaaagaattactgcaaaatggtatgaatggacgtaccat
attgggtagtgctttattagaagatgaatttacaccttttgatgttgttagacaatgctcaggtgttactttccaaagtgcagtgaaaagaacaatcaagggtacacaccact
ggttgttactcacaattttgacttcacttttagttttagtccagagtactcaatggtctttgttcttttttttgtatgaaaatgcctttttacctttgctatgggtattattgctatgtctg
cttttgcaatgatgtttgtcaaacataagcatgcatttctctgtttgtttttgttaccttctcttgccactgtagcttattttaatatggtctatatgcctgctagttgggtgatgcgta
ttatgacatggttggatatggttgatactagtttgtctggttttaagctaaaagactgtgttatgtatgcatcagctgtagtgttactaatccttatgacagcaagaactgtgtat

gatgatggtgctaggagagtgtggacacttatgaatgtcttgacactcgtttataaagtttattatggtaatgctttagatcaagccatttccatgtgggctcttataatctctg
ttacttctaactactcaggtgtagttacaactgtcatgtttttggccagaggtattgtttttatgtgtgttgagtattgccctattttcttcataactggtaatacacttcagtgtata
atgctagtttattgtttcttaggctatttttgtacttgttactttggcctcttttgtttactcaaccgctactttagactgactcttggtgtttatgattacttagtttctacacaggagtt
tagatatatgaattcacagggactactcccacccaagaatagcatagatgccttcaaactcaacattaaattgttgggtgttggtggcaaaccttgtatcaaagtagccac
tgtacagtctaaaatgtcagatgtaaagtgcacatcagtagtcttactctcagttttgcaacaactcagagtagaatcatcatctaaattgtgggctcaatgtgtccagttac
acaatgacattctcttagctaaagatactactgaagcctttgaaaaaatggtttcactactttctgttttgctttccatgcagggtgctgtagacataaacaagctttgtgaag
aaatgctggacaacagggcaaccttacaagctatagcctcagagtttagttcccttccatcatatgcagcttttgctactgctcaagaagcttatgagcaggctgttgcta
atggtgattctgaagttgttcttaaaaagttgaagaagtctttgaatgtggctaaatctgaatttgaccgtgatgcagccatgcaacgtaagttggaaaagatggctgatca
agctatgacccaaatgtataaacaggctagatctgaggacaagagggcaaaagttactagtgctatgcagacaatgcttttcactatgcttagaaagttggataatgatg
cactcaacaacattatcaacaatgcaagagatggttgtgttcccttgaacataatacctcttacaacagcagccaaactaatggttgtcataccagactataacacatata
aaaatacgtgtgatggtacaacatttacttatgcatcagcattgtgggaaatccaacaggttgtagatgcagatagtaaaattgttcaacttagtgaaattagtatggacaa
ttcacctaatttagcatggcctcttattgtaacagctttaagggccaattctgctgtcaaattacagaataatgagcttagtcctgttgcactacgacagatgtcttgtgctgc
cggtactacacaaactgcttgcactgatgacaatgcgttagcttactacaacacaacaaagggaggtaggtttgtacttgcactgttatccgatttacaggatttgaaatg
ggctagattccctaagagtgatggaactggtactatctatacagaactggaaccaccttgtaggtttgttacagacacacctaaaggtcctaaagtgaagtatttatacttt
attaaaggattaaacaacctaaatagaggtatggtacttggtagtttagctgccacagtacgtctacaagctggtaatgcaacagaagtgcctgccaattcaactgtatta
tctttctgtgcttttgctgtagatgctgctaaagcttacaaagattatctagctagtgggggacaaccaatcactaattgtgttaagatgttgtgtacacacactggtactggt
caggcaataacagttacaccggaagccaatatggatcaagaatcctttggtggtgcatcgtgttgtctgtactgccgttgccacatagatcatccaaatcctaaaggattt
tgtgacttaaaaggtaagtatgtacaaatacctacaacttgtgctaatgaccctgtgggttttacacttaaaaacacagtctgtaccgtctgcggtatgtggaaaggttatg
gctgtagttgtgatcaactccgcgaacccatgcttcagtcagctgatgcacaatcgtttttaaacgggtttgcggtgtaagtgcagcccgtcttacaccgtgcggcacag
gcactagtactgatgtcgtatacagggcttttgacatctacaatgataaagtagctggttttgctaaattcctaaaaactaattgttgtcgcttccaagaaaaggacgaagat
gacaatttaattgattcttactttgtagttaagagacacactttctctaactaccaacatgaagaaacaatttataatttacttaaggattgtccagctgttgctaaacatgactt
ctttaagtttagaatagacggtgacatggtaccacatatatcacgtcaacgtcttactaaatacacaatggcagacctcgtctatgctttaaggcattttgatgaaggtaatt
gtgacacattaaaagaaatacttgtcacatacaattgttgtgatgatgattatttcaataaaaaggactggtatgattttgtagaaaacccagatatattacgcgtatacgcc
aacttaggtgaacgtgtacgccaagctttgttaaaaacagtacaattctgtgatgccatgcgaaatgctggtattgttggtgtactgacattagataatcaagatctcaatg
gtaactggtatgatttcggtgatttcatacaaaccacgccaggtagtggagttcctgttgtagattcttattattcattgttaatgcctatattaaccttgaccagggctttaact
gcagagtcacatgttgacactgacttaacaaagccttacattaagtgggatttgttaaaatatgacttcacggaagagaggttaaaactctttgaccgttattttaaatattg
ggatcagacataccacccaaattgtgttaactgtttggatgacagatgcattctgcattgtgcaaactttaatgttttattctctacagtgttcccacctacaagttttggacca
ctagtgagaaaaatatttgttgatggtgttccatttgtagtttcaactggataccacttcagagagctaggtgttgtacataatcaggatgtaaacttacatagctctagactt
agttttaaggaattacttgtgtatgctgctgaccctgctatgcacgctgcttctggtaatctattactagataaacgcactacgtgcttttcagtagctgcacttactaacaatg
ttgcttttcaaactgtcaaacccggtaattttaacaaagacttctatgactttgctgtgtctaagggtttctttaaggaaggaagttctgttgaattaaaacacttcttctttgctc
aggatggtaatgctgctatcagcgattatgactactatcgttataatctaccaacaatgtgtgatatcagacaactactatttgtagttgaagttgttgataagtactttgattgt
tacgatggtggctgtattaatgctaaccaagtcatcgtcaacaacctagacaaatcagctggttttccatttaataaatggggtaaggctagactttattatgattcaatgag
ttatgaggatcaagatgcacttttcgcatatacaaaacgtaatgtcatccctactataactcaaatgaatcttaagtatgccattagtgcaaagaatagagctcgcaccgta
gctggtgtctctatctgtagtactatgaccaatagacagtttcatcaaaaattattgaaatcaatagccgccactagaggagctactgtagtaattggaacaagcaaattct
atggtggttggcacaacatgttaaaaactgtttatagtgatgtagaaaaccctcaccttatgggttgggattatcctaaatgtgatagagccatgcctaacatgcttagaatt
atggcctcacttgttcttgctcgcaaacatacaacgtgttgtagcttgtcacaccgtttctatagattagctaatgagtgtgctcaagtattgagtgaaatggtcatgtgtggc
ggttcactatatgttaaaccaggtggaacctcatcaggagatgccacaactgcttatgctaatagtgtttttaacatttgtcaagctgtcacggccaatgttaatgcactttta
tctactgatggtaacaaaattgccgataagtatgtccgcaatttacaacacagactttatgagtgtctctatagaaatagagatgttgacacagactttgtgaatgagtttta
cgcatatttgcgtaaacatttctcaatgatgatactctctgacgatgctgttgtgtgtttcaatagcacttatgcatctcaaggtctagtggctagcataaagaactttaagtca
gttctttattatcaaaacaatgtttttatgtctgaagcaaaatgttggactgagactgaccttactaaaggacctcatgaattttgctctcaacatacaatgctagttaaacagg
gtgatgattatgtgtaccttccttacccagatccatcaagaatcctaggggccggctgttttgtagatgatatcgtaaaaacagatggtacacttatgattgaacggttcgt
gtctttagctatagatgcttacccacttactaaacatcctaatcaggagtatgctgatgtctttcatttgtacttacaatacataagaaagctacatgatgagttaacaggaca
catgttagacatgtattctgttatgcttactaatgataacacttcaaggtattgggaacctgagttttatgaggctatgtacacaccgcatacagtcttacaggctgttgggg
cttgtgttctttgcaattcacagacttcattaagatgtggtgcttgcatacgtagaccattcttatgttgtaaatgctgttacgaccatgtcatatcaacatcacataaattagtc
ttgtctgttaatccgtatgtttgcaatgctccaggttgtgatgtcacagatgtgactcaactttacttaggaggtatgagctattattgtaaatcacataaaccacccattagttt
tccattgtgtgctaatggacaagtttttggtttatataaaaatacatgtgttggtagcgataatgttactgactttaatgcaattgcaacatgtgactggacaaatgctggtgat
tacattttagctaacacctgtactgaaagactcaagctttttgcagcagaaacgctcaaagctactgaggagacatttaaactgtcttatggtattgctactgtacgtgaagt
gctgtctgacagagaattacatctttcatgggaagttggtaaacctagaccaccacttaaccgaaattatgtctttactggttatcgtgtaactaaaaacagtaaagtacaa
ataggagagtacacctttgaaaaaggtgactatggtgatgctgttgtttaccgaggtacaacaacttacaaattaaatgttggtgattattttgtgctgacatcacatacagt
aatgccattaagtgcacctacactagtgccacaagagcactatgttagaattactggcttatacccaacactcaatatctcagatgagttttctagcaatgttgcaaattatc
aaaaggttggtatgcaaaagtattctacactccagggaccacctggtactggtaagagtcattttgctattggcctagctctctactacccttctgctcgcatagtgtataca

gcttgctctcatgccgctgttgatgcactatgtgagaaggcattaaaatatttgcctatagataaatgtagtagaattatacctgcacgtgctcgtgtagagtgttttgataaa
ttcaaagtgaattcaacattagaacagtatgtcttttgtactgtaaatgcattgcctgagacgacagcagatatagttgtctttgatgaaatttcaatggccacaaattatgatt
tgagtgttgtcaatgccagattacgtgctaagcactatgtgtacattggcgaccctgctcaattacctgcaccacgcacattgctaactaagggcacactagaaccagaa
tatttcaattcagtgtgtagacttatgaaaactataggtccagacatgttcctcggaacttgtcggcgttgtcctgctgaaattgttgacactgtgagtgctttggtttatgata
ataagcttaaagcacataaagacaaatcagctcaatgctttaaaatgttttataagggtgttatcacgcatgatgtttcatctgcaattaacaggcccacaaataggcgtggt
aagagaattccttacacgtaaccctgcttggagaaaagctgtctttatttcaccttataattcacagaatgctgtagcctcaaagattttgggactaccaactcaaactgttg
attcatcacagggctcagaatatgactatgtcatattcactcaaaccactgaaacagctcactcttgtaatgtaaacagatttaatgttgctattaccagagcaaaagtagg
catactttgcataatgtctgatagagacctttatgacaagttgcaatttacaagtcttgaaattccacgtaggaatgtggcaactttacaagctgaaaatgtaacaggactC
tttaaagattgtagtaaggtaatcactgggttacatcctacacaggcacctacacacctcagtgttgacactaaattcaaaactgaaggtttatgtgttgacatacctggca
tacctaaggacatgacctatagaagactcatctctatgatgggttttaaaatgaattatcaagttaatggttaccctaacatgtttatcacccgcgaagaagctataagacat
gtacgtgcatggattggcttcgatgtcgaggggtgtcatgctactagagaagctgttggtaccaatttacctttacagctaggtttttctacaggtgttaacctagttgctgt
acctacaggttatgttgatacacctaataatacagatttttccagagttagtgctaaaccaccgcctggagatcaatttaaacacctcataccacttatgtacaaaggacttc
cttggaatgtagtgcgtataaagattgtacaaatgttaagtgacacacttaaaaatctctctgacagagtcgtatttgtcttatgggcacatggctttgagttgacatctatga
agtattttgtgaaaataggacctgagcgcacctgttgtctatgtgatagacgtgccacatgcttttccactgcttcagacacttatgcctgttggcatcattctattggatttga
ttacgtctataatccgtttatgattgatgttcaacaatggggttttacaggtaacctacaaagcaaccatgatctgtattgtcaagtccatggtaatgcacatgtagctagttg
tgatgcaatcatgactaggtgtctagctgtccacgagtgctttgttaagcgtgttgactggactattgaatatcctataattggtgatgaactgaagattaatgcggcttgta
gaaaggttcaacacatggttgttaaagctgcattattagcagacaaattcccagttcttcacgacattggtaaccctaaagctattaagtgtgtacctcaagctgatgtaga
atggaagttctatgatgcacagccttgtagtgacaaagcttataaaatagaagaattattctattcttatgccacacattctgacaaattcacagatggtgtatgcctattttg
gaattgcaatgtcgatagatatcctgctaattccattgtttgtagatttgacactagagtgctatctaaccttaacttgcctggttgtgatggtggcagtttgtatgtaaataaa
catgcattccacacaccagcttttgataaaagtgcttttgttaatttaaaacaattaccatttttctattactctgacagtccatgtgagtctcatggaaaacaagtagtgtcag
atatagattatgtaccactaaagtctgctacgtgtataacacgttgcaatttaggtggtgctgtctgtagacatcatgctaatgagtacagattgtatctcgatgcttataaca
tgatgatctcagctggctttagcttgtgggtttacaaacaatttgatacttataacctctggaacacttttacaagacttcagagtttagaaaatgtggcttttaatgttgtaaat
aagggacactttgatggacaacagggtgaagtaccagtttctatcattaataacactgtttacacaaaagttgatggtgttgatgtagaattgtttgaaaataaaacaacatt
acctgttaatgtagcatttgagctttgggctaagcgcaacattaaaccagtaccagaggtgaaaatactcaataatttgggtgtggacattgctgctaatactgtgatctgg
gactacaaaagagatgctccagcacatatatctactattggtgtttgttctatgactgacatagccaagaaaccaactgaaacgatttgtgcaccactcactgtctttttga
tggtagagttgatggtcaagtagacttatttagaaatgcccgtaatggtgttcttattacagaaggtagtgttaaaggtttacaaccatctgtaggtcccaaacaagctagt
cttaatggagtcacattaattggagaagccgtaaaaacacagttcaattattataagaaagttgatggtgttgtccaacaattacctgaaacttactttactcagagtagaaa
tttacaagaatttaaacccaggagtcaaatggaaattgatttcttagaattagctatggatgaattcattgaacggtataaattagaaggctatgccttcgaacatatcgttta
tggagattttagtcatagtcagttaggtggtttacatctactgattggactagctaaacgttttaaggaatcaccttttgaattagaagattttattcctatggacagtacagtta
aaaactatttcataacagatgcgcaaacaggttcatctaagtgtgtgtgttctgttattgatttattacttgatgattttgttgaaataataaaatcccaagatttatctgtagtttc
taaggttgtcaaagtgactattgactatacagaaatttcatttatgctttggtgtaaagatggccatgtagaaacattttacccaaaattacaatctagtcaagcgtggcaac
cgggtgttgctatgcctaatctttacaaaatgcaaagaatgctattagaaaagtgtgaccttcaaaattatggtgatagtgcaacattacctaaaggcataatgatgaatgt
cgcaaaatatactcaactgtgtcaatatttaaacacattaacattagctgtaccctataatatgagagttatacattttggtgctggttctgataaaggagttgcaccaggtac
agctgttttaagacagtggttgcctacgggtacgctgcttgtcgattcagatcttaatgactttgtctctgatgcagattcaactttgattggtgattgtgcaactgtacataca
gctaataaatgggatctcattattagtgatatgtacgaccctaagactaaaaatgttacaaaagaaaatgactctaaagagggtttttcacttacatttgtgggtttatacaa
caaaagctagctcttggaggttccgtggctataaagataacagaacattcttggaatgctgatctttataagctcatgggacacttcgcatggtggacagcctttgttacta
atgtgaatgcgtcatcatctgaagcatttttaattggatgtaattatcttggcaaaccacgcgaacaaatagatggttatgtcatgcatgcaaattacatattttggaggaata
caaatccaattcagttgtcttcctattctttatttgacatgagtaaatttccccttaaattaaggggtactgctgttatgtctttaaaagaaggtcaaatcaatgatatgattttatc
tcttcttagtaaaggtagacttataattagagaaaacaacagagttgttatttctagtgatgttcttgttaacaactaaacgaacaatgtttgtttttcttgttttattgccactagt
ctctagtcagtgtgttaatcttacaaccagaactcaattaccccctgcatacactaattctttcacacgtggtgtttattaccctgacaaagttttcagatcctcagttttacatt
caactcaggacttgttcttacctttcttttccaatgttacttggttccatgctatacatgtctctgggaccaatggtactaagaggtttgataaccctgtcctaccatttaatgat
ggtgtttattttgcttccactgagaagtctaacataataagaggctggatttttggtactactttagattcgaagacccagtccctacttattgttaataacgctactaatgttgt
tattaaagtctgtgaatttcaattttgtaatgatccatttttgggtgtttattaccacaaaaacaacaaaagttggatggaaagtgagttcagagtttattctagtgcgaataatt
gcacttttgaatatgtctctcagccttttcttatggaccttgaaggaaaacagggtaatttcaaaaatcttagggaatttgtgtttaagaatattgatggttattttaaaatatatt
ctaagcacacgcctattaatttagtgcgtgatctccctcagggtttttcggctttagaaccattggtagatttgccaataggtattaacatcactaggtttcaaactttacttgc
tttacatagaagttatttgactcctggtgattcttcttcaggttggacagctggtgctgcagcttattatgtgggttatcttcaacctaggacttttctattaaaatataatgaaaa
tggaaccattacagatgctgtagactgtgcacttgaccctctctcagaaacaaagtgtacgttgaaatccttcactgtagaaaaaggaatctatcaaacttctaactttaga
gtccaaccaacagaatctattgttagatttcctaatattacaaacttgtgcccttttggtgaagtttttaacgccaccagatttgcatctgtttatgcttggaacaggaagaga
atcagcaactgtgttgctgattattctgtcctatataattccgcatcattttccacttttaagtgttatggagtgtctcctactaaattaaatgatctctgctttactaatgtctatgc
agattcatttgtaattagaggtgatgaagtcagacaaatcgctccagggcaaactggaaagattgctgattataattataaattaccagatgattttacaggctgcgttata
gcttggaattctaacaatcttgattctaaggttggtggtaattataattacctgtatagattgtttaggaagtctaatctcaaaccttttgagagagatatttcaactgaaatctat

caggccggtagcacaccttgtaatggtgttgaaggttttaattgttactttcctttacaatcatatggtttccaacccactaatggtgttggttaccaaccatacagagtagta
gtactttcttttgaacttctacatgcaccagcaactgtttgtggacctaaaaagtctactaatttggttaaaaacaaatgtgtcaatttcaacttcaatggtttaacaggcacag
gtgttcttactgagtctaacaaaaagtttctgcctttccaacaatttggcagagacattgctgacactactgatgctgtccgtgatccacagacacttgagattcttgacatta
caccatgttcttttggtggtgtcagtgttataacaccaggaacaaatacttctaaccaggttgctgttctttatcaggatgttaactgcacagaagtccctgttgctattcatg
cagatcaacttactcctacttggcgtgtttattctacaggttctaatgtttttcaaacacgtgcaggctgtttaataggggctgaacatgtcaacaactcatatgagtgtgaca
tacccattggtgcaggtatatgcgctagttatcagactcagactaattctcctcggcgggcacgtagtgtagctagtcaatccatcattgcctacactatgtcacttggtgc
agaaaattcagttgcttactctaataactctattgccatacccacaaattttactattagtgttaccacagaaattctaccagtgtctatgaccaagacatcagtagattgtac
aatgtacatttgtggtgattcaactgaatgcagcaatcttttgttgcaatatggcagtttttgtacacaattaaaccgtgctttaactggaatagctgttgaacaagacaaaaa
cacccaagaagtttttgcacaagtcaaacaaatttacaaaacaccaccaattaaagattttggtggttttaatttttcacaaatattaccagatccatcaaaaccaagcaag
aggtcatttattgaagatctacttttcaacaaagtgacacttgcagatgctggcttcatcaaacaatatggtgattgccttggtgatattgctgctagagacctcatttgtgca
caaaagtttaacggccttactgttttgccacctttgctcacagatgaaatgattgctcaatacacttctgcactgttagcgggtacaatcacttctggttggacctttggtgca
ggtgctgcattacaaataccatttgctatgcaaatggcttataggtttaatggtattggagttacacagaatgttctctatgagaaccaaaaattgattgccaaccaatttaat
agtgctattggcaaaattcaagactcactttcttccacagcaagtgcacttggaaaacttcaagatgtggtcaaccaaaatgcacaagctttaaacacgcttgttaaacaa
cttagctccaattttggtgcaatttcaagtgttttaaatgatatcctttcacgtcttgacaaagttgaggctgaagtgcaaattgataggttgatcacaggcagacttcaaagt
ttgcagacatatgtgactcaacaattaattagagctgcagaaatcagagcttctgctaatcttgctgctactaaaatgtcagagtgtgtacttggacaatcaaaaagagttg
atttttgtggaaagggctatcatcttatgtccttccctcagtcagcacctcatggtgtagtcttcttgcatgtgacttatgtccctgcacaagaaaagaacttcacaactgctc
ctgccatttgtcatgatggaaaagcacactttcctcgtgaaggtgtctttgtttcaaatggcacacactggtttgtaacacaaaggaatttttatgaaccacaaatcattacta
cagacaacacatttgtgtctggtaactgtgatgttgtaataggaattgtcaacaacacagtttatgatcctttgcaacctgaattagactcattcaaggaggagttagataaa
tattttaagaatcatacatcaccagatgttgatttaggtgacatctctggcattaatgcttcagttgtaaacattcaaaaagaaattgaccgcctcaatgaggttgccaagaa
tttaaatgaatctctcatcgatctccaagaacttggaaagtatgagcagtatataaaatggccatggtacatttggctaggttttatagctggcttgattgccatagtaatggt
gacaattatgctttgctgtatgaccagttgctgtagttgtctcaagggctgttgttcttgtggatcctgctgcaaatttgatgaagacgactctgagccagtgctcaaagga
gtcaaattacattacacataaacgaacttatggatttgtttatgagaatcttcacaattggaactgtaactttgaagcaaggtgaaatcaaggatgctactccttcagattttg
ttcgcgctactgcaacgataccgatacaagcctcactccctttcggatggcttattgttggcgttgcacttcttgctgtttttcagagcgcttccaaaatcataaccctcaaa
aagagatggcaactagcactctccaagggtgttcactttgtttgcaacttgctgttgttgtttgtaacagtttactcacaccttttgctcgttgctgctggccttgaagcccctt
ttctctatctttatgctttagtctacttcttgcagagtataaactttgtaagaataataatgaggctttggctttgctggaaatgccgttccaaaaacccattactttatgatgcca
actattttctttgctggcatactaattgttacgactattgtataccttacaatagtgtaacttcttcaattgtcattacttcaggtgatggcacaacaagtcctatttctgaacatg
actaccagattggtggttatactgaaaaatgggaatctggagtaaaagactgtgttgtattacacagttacttcacttcagactattaccagctgtactcaactcaattgagt
acagacactggtgttgaacatgttaccttcttcatctacaataaaattgttgatgagcctgaagaacatgtccaaattcacacaatcgacggttcatccggagttgttaatcc
agtaatggaaccaatttatgatgaaccgacgacgactactagcgtgcctttgtaagcacaagctgatgagtacgaacttatgtactcattcgtttcggaagagacaggta
cgttaatagttaatagcgtacttctttttcttgctttcgtggtattcttgctagttacactagccatccttactgcgcttcgattgtgtgcgtactgctgcaatattgttaacgtgag
tcttgtaaaaccttctttttacgtttactctcgtgttaaaaatctgaattcttctagagttcctgatcttctggtctaaacgaactaaatattatattagtttttctgtttggaactttaa
ttttagccatggcagattccaacggtactattaccgttgaagagcttaaaaagctccttgaacaatggaacctagtaataggtttcctattccttacatggatttgtcttctac
aatttgcctatgccaacaggaataggtttttgtatataattaagttaattttcctctggctgttatggccagtaactttagcttgttttgtgcttgctgctgtttacagaataaattg
gatcaccggtggaattgctatcgcaatggcttgtcttgtaggcttgatgtggctcagctacttcattgcttctttcagactgtttgcgcgtacgcgttccatgtggtcattcaa
tccagaaactaacattcttctcaacgtgccactccatggcactattctgaccagaccgcttctagaaagtgaactcgtaatcggagctgtgatccttcgtggacatcttcgt
attgctggacaccatctaggacgctgtgacatcaaggacctgcctaaagaaatcactgttgctacatcacgaacgctttcttattacaaattgggagcttcgcagcgtgta
gcaggtgactcaggttttgctgcatacagtcgctacaggattggcaactataaattaaacacagaccattccagtagcagtgacaatattgctttgcttgtacagtaagtg
acaacagatgtttcatctcgttgactttcaggttactatagcagagatattactaattattatgaggacttttaaagtttccatttggaatcttgattacatcataaacctcataatt
aaaaatttatctaagtcactaactgagaataaatattctcaattagatgaagagcaaccaatggagattgattaaacgaacATGGTGAGCAAAGGAGA
AGAGGACAACATGGCATCACTCCCAGCTACACATGAGCTGCATATCTTCGGATCCATCAACGGAGT
GGACTTCGATATGGTGGGACAGGGTACAGGGAACCCAAACGACGGATACGAGGAGTTGAACCTGA
AGAGTACCAAGGGAGACCTGCAGTTCTCACCATGGATACTCGTCCCACATATAGGATACGGCTTTCA
TCAGTACCTGCCCTATCCAGACGGAATGTCACCTTTCCAGGCAGCCATGGTTGACGGGAGCGGTTAC
CAGGTCCACAGGACAATGCAGTTTGAGGACGGAGCCTCATTGACCGTGAACTACAGATATACCTAC
GAAGGAAGCCATATCAAGGGAGAGGCTCAAGTGAAGGGAACTGGATTCCCAGCGGACGGACCCGT
GATGACCAACAGTCTGACGGCTGCAGACTGGTGCAGATCCAAAAAGACCTACCCAAATGACAAGAC
AATCATAAGCACCTTCAAGTGGTCATACACTACAGGAAACGGGAAGAGATACAGGAGCACTGCCAG
AACCACATACACTTTCGCCAAGCCTATGGCTGCAAACTACCTCAAGAACCAACCCATGTATGTGTTC
AGAAAGACAGAACTGAAGCATTCTAAGACCGAACTGAACTTCAAGGAGTGGCAGAAGGCCTTTACT
GACGTGATGGGAATGGACGAACTCTACAAGTAATTAATTAAgaactttcattaattgacttctatttgtgctttttagcctttctgct
attccttgttttaattatgcttattatcttttggttctcacttgaactgcaagatcataatgaaacttgtcacgcctaaacgaacatgaaatttcttgttttcttaggaatcatcaca

actgtagctgcatttcaccaagaatgtagtttacagtcatgtactcaacatcaaccatatgtagttgatgacccgtgtcctattcacttctattctaaatggtatattagagtag
gagctagaaaatcagcacctttaattgaattgtgcgtggatgaggctggttctaaatcacccattcagtacatcgatatcggtaattatacagtttcctgttCacctttttaca
attaattgccaggaacctaaattgggtagtcttgtagtgcgttgttcgttctatgaagactttttagagtatcatgacgttcgtgttgttttagatttcatctaaacgaacaaact
aaaatgtctgataatggaccccaaaatcagcgaaatgcaccccgcattacgtttggtggaccctcagattcaactggcagtaaccagaatggagaacgcagtggggc
gcgatcaaaacaacgtcggccccaaggtttacccaataatactgcgtcttggttcaccgctctcactcaacatggcaaggaagaccttaaattccctcgaggacaagg
cgttccaattaacaccaatagcagtccagatgaccaaattggctactaccgaagagctaccagacgaattcgtggtggtgacggtaaaatgaaagatctcagtccaag
atggtatttctactacctaggaactgggccagaagctggacttccctatggtgctaacaaagacggcatcatatgggttgcaactgagggagccttgaatacaccaaaa
gatcacattggcacccgcaatcctgctaacaatgctgcaatcgtgctacaacttcctcaaggaacaacattgccaaaaggcttctacgcagaagggagcagaggcgg
cagtcaagcctcttctcgttcctcatcacgtagtcgcaacagttcaagaaattcaactccaggcagcagtaggggaacttctcctgctagaatggctggcaatggcggt
gatgctgctcttgctttgctgctgcttgacagattgaaccagcttgagagcaaaatgtctggtaaaggccaacaacaacaaggccaaactgtcactaagaaatctgctg
ctgaggcttctaagaagcctcggcaaaaacgtactgccactaaagcatacaatgtaacacaagctttcggcagacgtggtccagaacaaacccaaggaaattttggg
gaccaggaactaatcagacaaggaactgattacaaacattggccgcaaattgcacaatttgcccccagcgcttcagcgttcttcggaatgtcgcgcattggcatggaa
gtcacaccttcgggaacgtggttgacctacacaggtgccatcaaattggatgacaaagatccaaatttcaaagatcaagtcattttgctgaataagcatattgacgcata
caaaacattcccaccaacagagcctaaaaaggacaaaaagaagaaggctgatgaaactcaagccttaccgcagagacagaagaaacagcaaactgtgactcttctt
cctgctgcagatttggatgatttctccaaacaattgcaacaatccatgagcagtgctgactcaactcaggcctaaactcatgcagaccacacaaggcagatgggctata
taaacgttttcgcttttccgtttacgatatatagtctactcttgtgcagaatgaattctcgtaactacatagcacaagtagatgtagttaactttaatctcacatagcaatctttaa
tcagtgtgtaacattagggaggacttgaaagagccaccacattttcaccgaggccacgcggagtacgatcgagtgtacagtgaacaatgctagggagagctgcctat
atggaagagccctaatgtgtaaaattaattttagtagtgctatccccatgtgattttaatagcttcttaggagaatgacaaaaaaaaaaaaaaaaaaaaaaaaaaaaa

Supplementary Figure 1. Genome sequence of mNG SARS-CoV-2. The mNG ORF with additional PacI restriction enzyme sites are shown in red.

Supplementary Figure 1

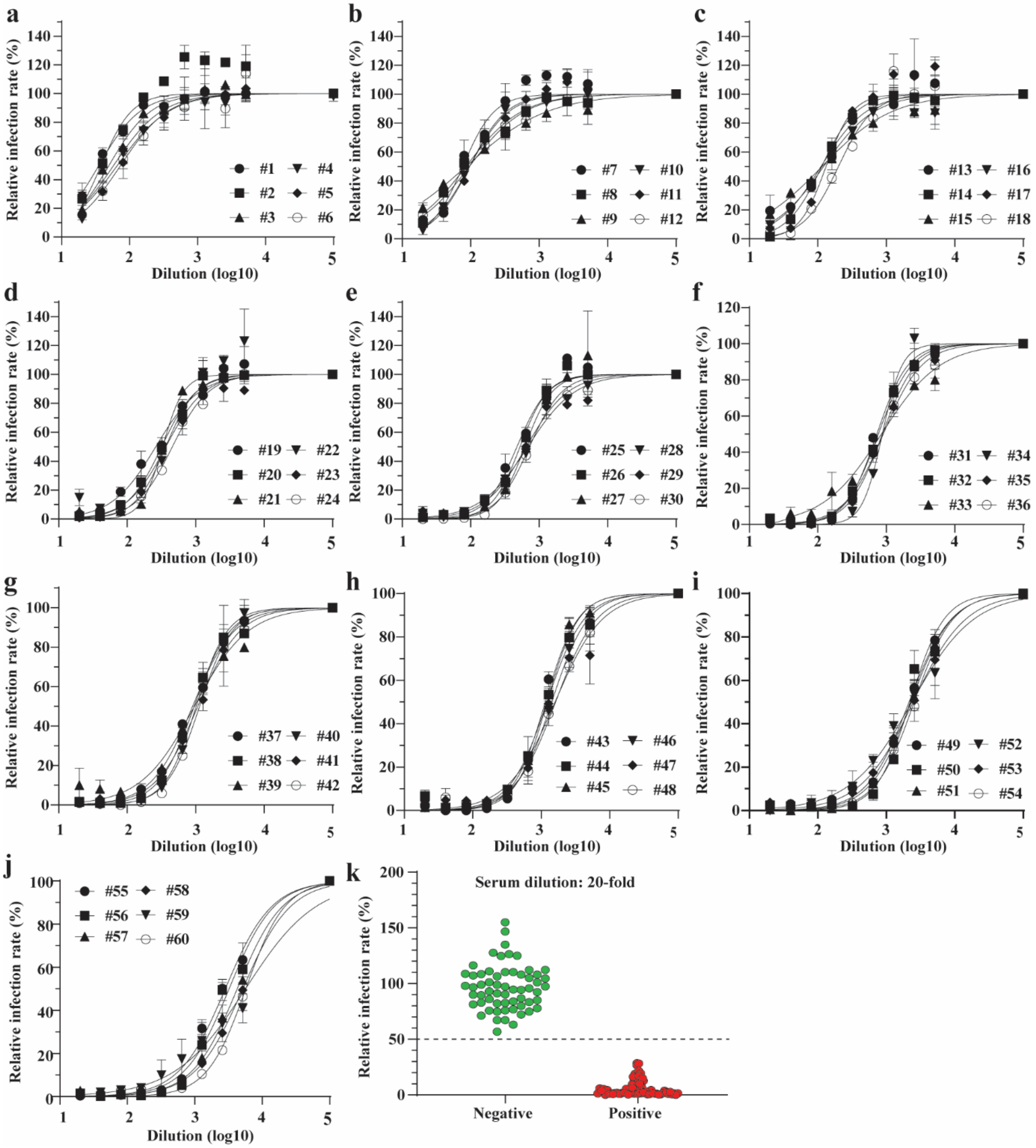

Supplementary Figure 2. **Analysis of neutralizing activities of human sera using mNG SARS-CoV-2.** (a-j) Neutralization curves for sixty specimens from patients confirmed with RT-PCR test positive. Means and standard deviations from two-independent experiments are shown. The dose-response curves were fitted using a nonlinear regression model in software Prism 8. (k) Relative infection rate of mNG SARS-CoV-2 for sixty COVID-19-positive and sixty COVID-19-negative human sera at dilution of 20 folds.
